# Supplementary material for: A STING-based biosensor affords broad cyclic dinucleotide detection within single living eukaryotic cells
Source: Nat Commun. 2020 Jul 15;11:3533. doi: 10.1038/s41467-020-17228-y (PMC7363834; doi:10.1038/s41467-020-17228-y)
Supplement: Supplementary file 1 — Supplementary Information [file 41467_2020_17228_MOESM1_ESM.pdf]

Supplementary Information

A STING-based Biosensor Affords Broad Cyclic Dinucleotide Detection Within Single Living Eukaryotic Cells

Pollock, Zaver, and Woodward

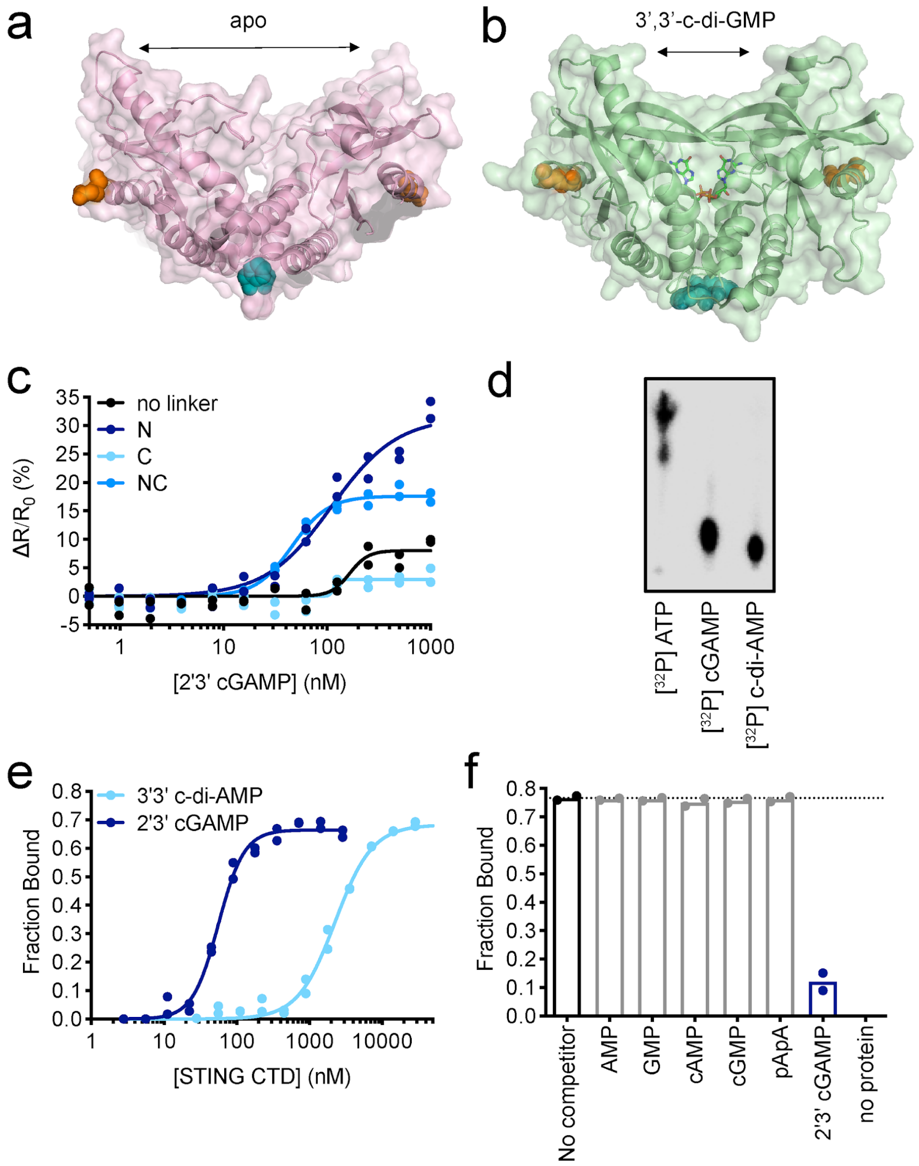

Supplementary Figure 1. BioSTING development and characterization: a-b STING crystal structures [40] with N terminus highlighted in Teal and C terminus highlighted in Orange where mTFP and mKO2 fluorophores were respectively attached (a) STING Apo structure (pink) (PDB 4F5E) and (b) STING bound to 3'3'-c-di-GMP (green) (PDB 4F5D) **c** FRET response in the presence of increasing 2'3'-cGAMP of prototype biosensor (black) and biosensors with GGSGG linkers on the N (dark blue), C (light blue), or both (medium blue) termini of STING-CTD **d** TLC analysis of enzymatically synthesized [^{32}P] labeled 2'3'-cGAMP and 3'3'-c-di-AMP and [^{32}P] ATP standard. Data are representative of two independent experiments **e** DRaCALA binding analysis of STING-CTD using ~1 nM [^{32}P] labeled 2'3'-cGAMP and 3'3'-c-di-AMP. **f** DRaCALA binding analysis of recombinant STING-CTD using ~1 nM [^{32}P] labeled 2'3'-cGAMP in the presence of excess (500 μM) unlabeled nucleotides. In all panels, individual data points of n=2 biological replicates are shown.

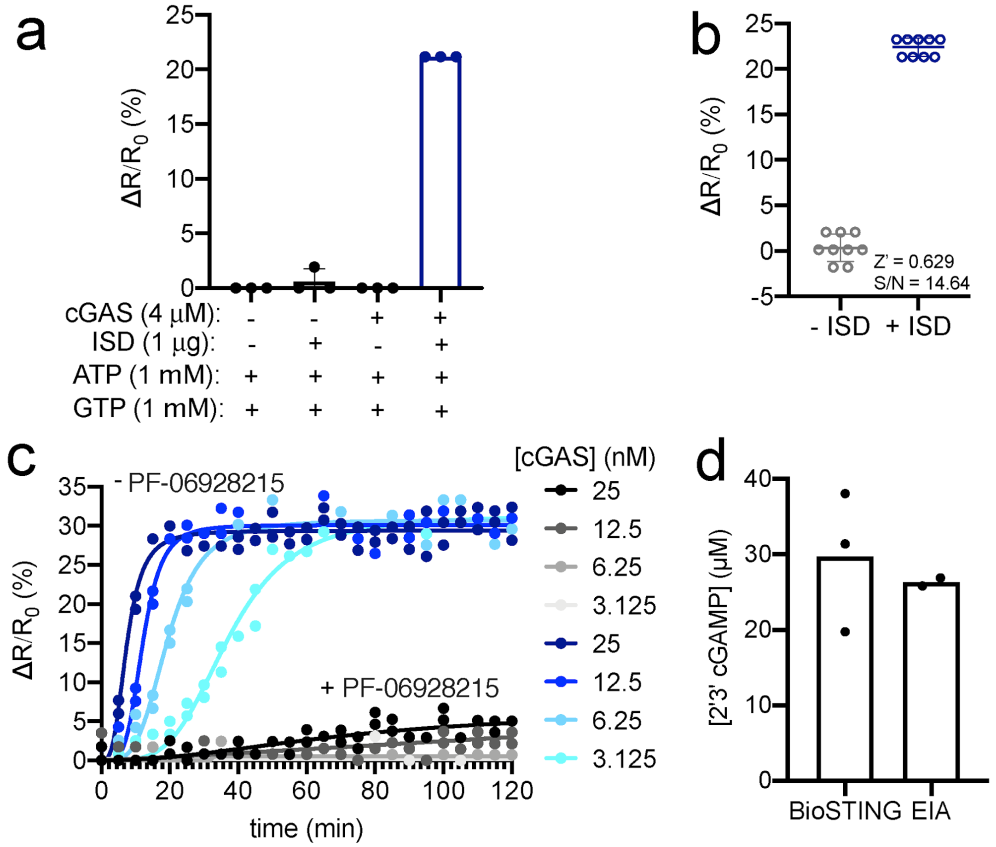

Supplementary Figure 2. BioSTING detection of CDNs *in vitro*. a cGAS activity assay in the presence and absence of indicated concentrations of recombinant cGAS, ISD, ATP, and GTP using BioSTING. **b** Determination of Z' factor and signal to noise (S/N) ratio for BioSTING using recombinant cGAS in the presence and absence of ISD in a 96-well format. 0.5 < Z' < 1 is considered excellent statistical reliability for high throughput screening applications. **c** cGAS activity assay measuring 2'3'-cGAMP production in the presence of a fixed concentration of PF-06928215 (500 µM) with increasing concentrations of cGAS using BioSTING. **d** Quantification of 2'3'-cGAMP levels from HEK293T cells transfected with 5 µg of pcDNA3.1-cGAS using BioSTING or EIA. In panels a and b, data are presented as mean ± s.d. of n=3 (a) or n=9 (b) biological replicates. In panels c and d, individual data points of n=2 (c, d) or n=3 (d) biological replicates are shown.

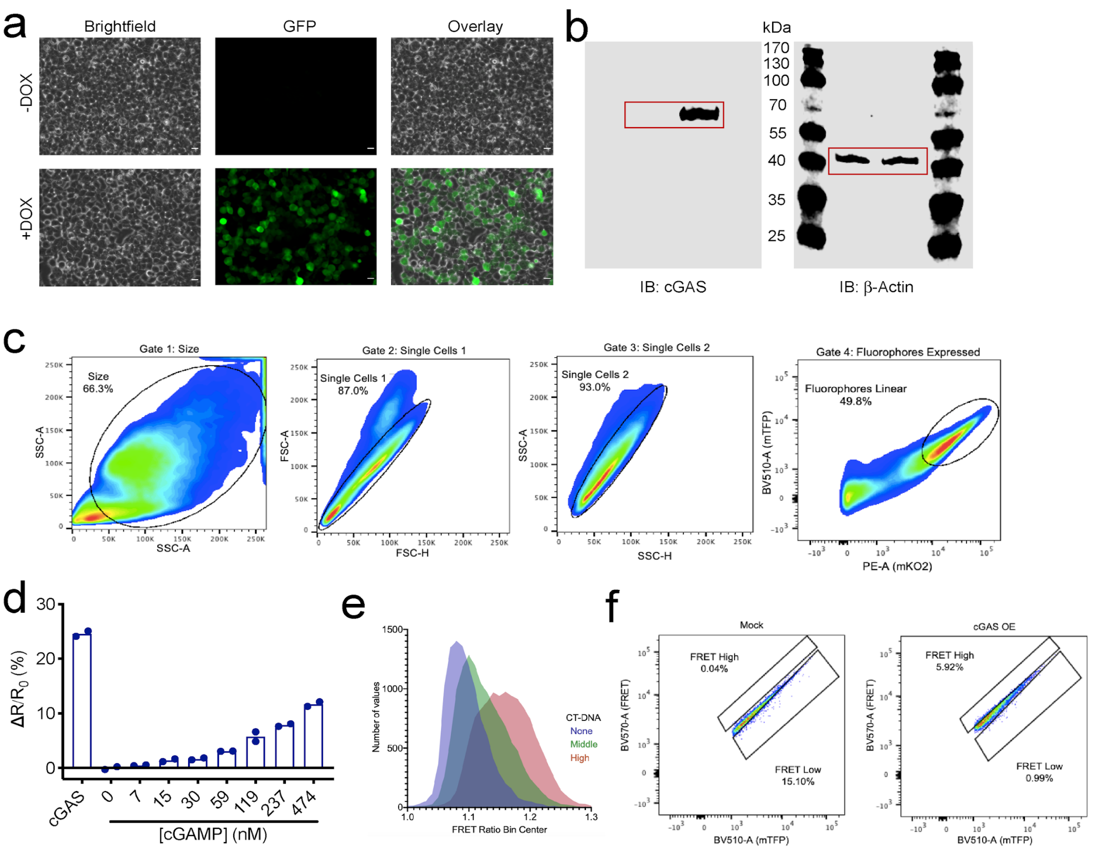

Supplementary Figure 3. BioSTING can detect cGAMP in cells. a BioSTING expression was induced in HEK293T cells transduced with pSLIK-BioSTING for 24 hours after the addition of doxycycline or vehicle control. Biosensor expression was analyzed using a BZ-X710 microscope (Keyence), GFP filter cube (EX 470/40, DM 495, and BA 525/50), and 40X objective. Scale bars, 15 µm. Data are representative of two independent experiments. **b** Uncropped blots for Fig. 3a of HEK293T cells stably expressing BioSTING transfected with either 1 µg of empty-pCDNA3.1 vector or pCDNA3.1-cGAS vector. Data are representative of two independent experiments. **c** Flow analysis method: Step 1 is to set a permissive size gate, Step 2 and 3 are sorting for single cells using FSC and SSC A by H plots, and step 4 is to plot BV510 (mTFP) by PE (mKO2) and gate on cells with appropriate expression. These cells are then analyzed for BV570 (FRET)/BV510 (CFP) ratio. This method was used to collect data for all flow analysis unless otherwise noted **d** HEK293T cells stably expressing BioSTING were transfected with either pCDNA3.1-cGAS or increasing concentrations of purified 2'3'-cGAMP using Lipofectamine 2000 transfection reagent according to the manufacturer's protocol and analyzed for FRET response by flow cytometry. Individual data points of n=2 biological replicates are shown. **e** Single cell analysis of HEK293T cells stably expressing BioSTING transfected with 10 ng of pCDNA3.1-cGAS and then transfected with a mock (0 ng), medium (156 ng), or high (1250 ng) amounts of cGAS-activating CT-DNA for 4 hours and analyzed by flow cytometry. **f** FRET high and low gates made on cells gated by above method (c) used to demonstrate flow selection potential. (Left) cells transfected with empty vector (Right) cells transfected with pCDNA3.1-cGAS. This method was used to collect data for figures 3g and 3h.

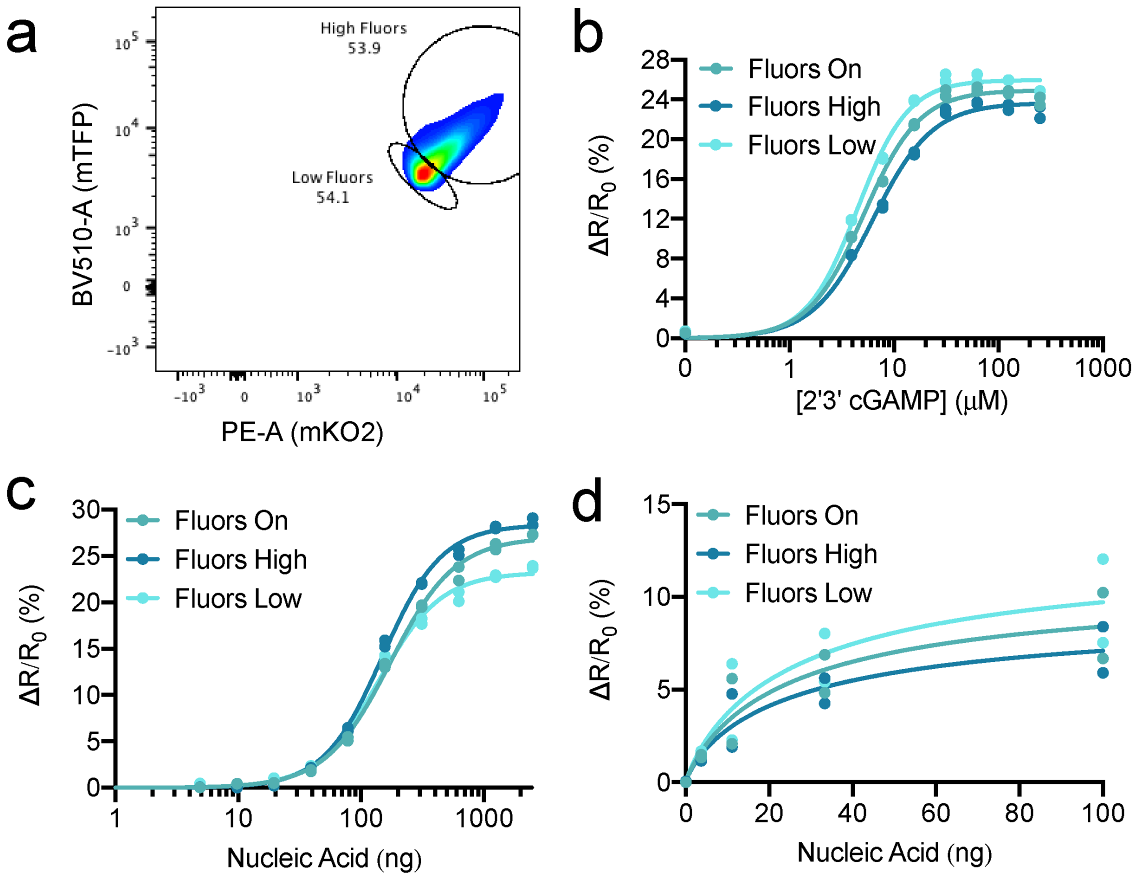

Supplementary Figure 4. Effects of BioSTING expression levels on FRET responses. a Representative flow plot demonstrating the gating method for separation of high and low BioSTING expressing populations. Cells were first analyzed as in supplemental figure 3c and then separated as shown. This method was used to collect data for all figures in this panel. **b** Data from figure 3d was reanalyzed to compare FRET responses from populations of cells expressing high and low levels of BioSTING **c** Data from figure 5a was reanalyzed to compare FRET responses from populations of cells expressing high and low levels of BioSTING. **d** Data from figure 5c was reanalyzed to compare FRET responses from populations of cells expressing high and low levels of BioSTING. In all panels, individual data points of n=2 biological replicates are shown.

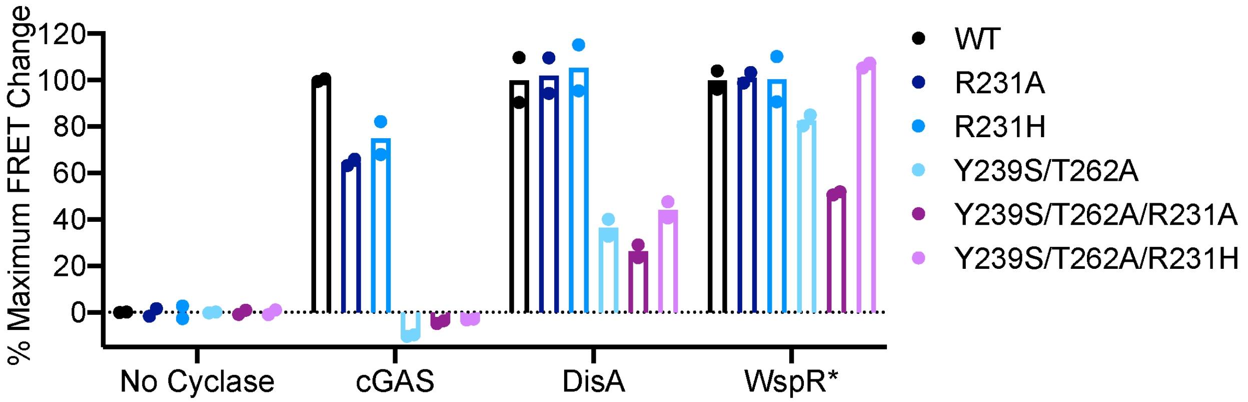

Supplementary Figure 5. BioSTING R231 Mutations. HEK293T cells stably expressing WT, R231A, R231H, Y239S/T262A, R231A/Y239S/T262A, or R231H/Y239S/T262A BioSTING were transfected with expression vectors for cGAS, DisA, WspR*, or empty vector and analyzed for FRET response by flow cytometry. Individual data points of n=2 biological replicates are shown.

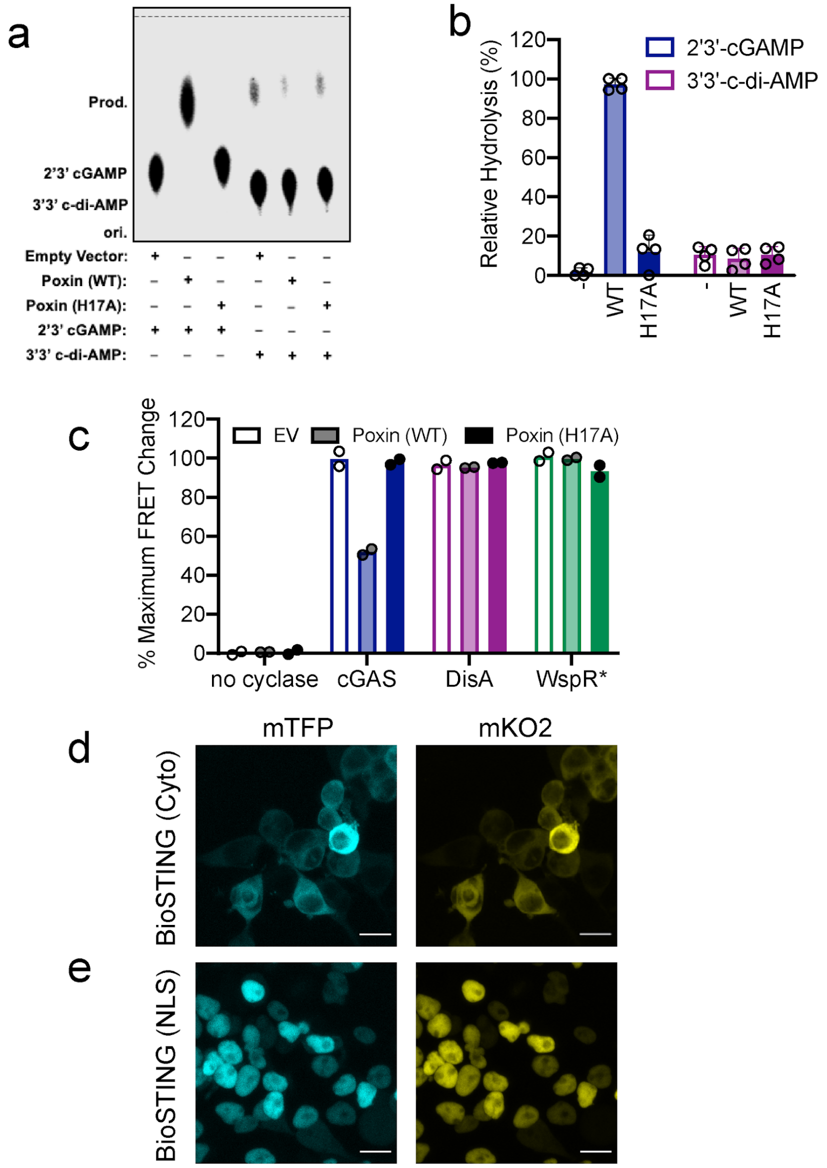

Supplementary Figure 6. Poxin Activity Assays and BioSTING localization. a TLC analysis of [^{32}P] labeled 2'3'-cGAMP and 3'3'-c-di-AMP degradation following 1h incubation in lysates from HEK293T cells transfected with 3 µg of empty vector or plasmid expressing wildtype (WT) or catalytically-dead (H17A) Poxin. Data are representative of four independent experiments. Relative hydrolysis from four biologically, independent samples is quantified on the right in panel **b**. Data are presented as mean ± s.d. of n=4 biological replicates. **c** HEK293T cells stably expressing BioSTING were transfected with 3 µg of empty vector or plasmid expressing WT or H17A Poxin along with 40 ng of plasmid expressing cGAS, DisA, or WspR* and analyzed for FRET response by flow cytometry. Individual data points of n=2 biological replicates are shown. **d-e** BioSTING expression was induced in HEK293T cells transduced with pSLIK-BioSTING (d) or pSLIK-NLS-BioSTING (e) for 24 hours by the addition of doxycycline and biosensor expression was analyzed by confocal microscopy using a Leica SP8X confocal microscope (Leica). Scale bars, 15 µm. Data are representative of two independent experiments.

Supplementary Table 1. BioSTING Parameters

BioSTING Parameters	
Kd (nM)	56
EC50 (nM)	77.4
L.O.D (nM)	12.6
L.O.Q. (nM)	38.2
Dynamic Range (nM)	12-125
∆FRET Ratio (%)	~25

Supplementary Table 2. Primers used in this study

Primer	Use	Sequence
1	Forward Amplify STING CTD no linker	GAGGAGACTAGTTTAAATGTTGCCCACGGGCTG
2	Reverse Amplify STING CTD no linker	GAGGAGGGTACCTTCCTGACGAATGTGCCGGAG
3	Forward Amplify STING CTD GGSGG linker	GAGGAGACTAGTGGCGGATCCGGGGGCTTAAATGTTGCCCACGGGCTG
4	Reverse Amplify STING CTD GGSGG linker	GAGGAGGGTACCGCCCCCGGATCCGCCTTCCTGACGAATGTGCCGGAG
5	Forward Y239S QuickChange	GGCATCAAGAATCGGGTTTCTTCCAACAGCGTCTACGAG
6	Reverse Y239S QuickChange	CTCGTAGACGCTGTTGGAAGAAACCCGATTCTTGATGCC
7	Forward T262A QuickChange	CTGTATCCTGGAGTACGCCGCCCCCTTGCAGACCC
8	Reverse T262A QuickChange	GGGTCTGCAAGGGGGCGGCGTACTCCAGGATACAG
9	Forward R231H QuickChange	CCCAGCAAAACATCGACCATGCTGGCATCAAGAATCGG
10	Reverse R231H QuickChange	CCGATTCTTGATGCCAGCATGGTCGATGTTTTGCTGGG
11	Forward R231A QuickChange	CCCCAGCAAAACATCGACGCTGCTGGCATCAAGAATCGG

12	Reverse R231A QuickChange	CCGATTCTTGATGCCAGCAGCGTCGATGTTTTGCTGGGG
13	Forward Amplify BioSTING for pSLIK	TGATCACTAGCGTACGACCATGGGCAGCAGCCATCATC ATC
14	Reverse Amplify BioSTING for pSLIK	TCTTCCAATTCGTACGTCATCCGCCAAAACAGCCAAG
15	Reverse Amplify BioSTING for pSLIK with NLS tag	TCTTCCAATTCGTACGTCAGTCCAACTTGACCCTCTTGG CAGCAGGTCCGCCAAAACAGCCAAGC

Supplementary Table 3. Plasmids used in this study

Number	Name	Use	Source
1	pET15b-mKO2-12AA-mTFP	Contained FRET fluorophores	Gift from Samuel Miller
2	pET15b-BioSTING	Bacterial expression vector	This study
3	pET15b-BioSTING (Y239S T262A)	Bacterial expression vector	This study
4	pSLIK-Empty Vector	Eukaryotic expression vector	Gift from Andrew Oberst
5	pSLIK-BioSTING	Eukaryotic expression vector	This study
6	pSLIK-BioSTING (R231A)	Eukaryotic expression vector	This study
7	pSLIK-BioSTING (R231H)	Eukaryotic expression vector	This study
8	pSLIK-BioSTING (Y239S T262A)	Eukaryotic expression vector	This study
9	pSLIK-BioSTING (R231A Y239S T262A)	Eukaryotic expression vector	This study
10	pSLIK-BioSTING (R231H Y239S T262A)	Eukaryotic expression vector	This study
11	pSLIK-NLS BioSTING	Expresses nuclear localized BioSTING	This study
12	pSLIK-NLS BioSTING (Y239S T262A)	Expresses nuclear localized BioSTING (Y239S T262A)	This study
13	pcDNA3-empty vector	Vector Control in Eukaryotic cells	Gift from Genhong Cheng
14	pcDNA3-cGAS	Expresses full-length human cGAS in Eukaryotic cells	Gift from Genhong Cheng
15	pcDNA4-DisA	Expresses DisA in Eukaryotic cells	Gift from Philip Kranzusch

16	pcDNA4-WspR*	Expresses WspR* in Eukaryotic cells	Gift from Philip Kranzusch
17	pcDNA4-Poxin (WT)	Expresses Poxin (WT) in Eukaryotic cells	Gift from Philip Kranzusch
18	pcDNA4-Poxin (H17A)	Expresses Poxin (H17A) in Eukaryotic cells	Gift from Philip Kranzusch
19	pcDNA3-hSTING (FL)	Expresses hSTING in Eukaryotic cells	Gift from Genhong Cheng
20	pSPEEDET-mRECON	Bacterial expression vector	[71]
21	pet28-mSTING-CTD	Bacterial expression vector	Gift from Russell Vance
22	pet20b-DisA	Bacterial expression vector	[72]
23	pET28a-His6-SUMO-mcGAS	Bacterial expression vector	Gift from Russell Vance

Supplementary References:

71. McFarland, A. P. *et al.* Sensing of Bacterial Cyclic Dinucleotides by the Oxidoreductase RECON Promotes NF-κB Activation and Shapes a Proinflammatory Antibacterial State. *Immunity* **46**, 433–445 (2017).
72. Sureka, K. *et al*. The cyclic dinucleotide c-di-AMP is an allosteric regulator of metabolic enzyme function. *Cell* **158**, 1389–1401 (2014).
