# Supplementary material for: A STING-based biosensor affords broad cyclic dinucleotide detection within single living eukaryotic cells
Source: Nat Commun. 2020 Jul 15;11:3533. doi: 10.1038/s41467-020-17228-y (PMC7363834; doi:10.1038/s41467-020-17228-y)
Supplement: Supplementary file 3 — Reporting Summary [file 41467_2020_17228_MOESM3_ESM.pdf]

nature research

Corresponding author(s): Joshua Woodward

Last updated by author(s): Jun 2, 2020

Reporting Summary

Nature Research wishes to improve the reproducibility of the work that we publish. This form provides structure for consistency and transparency in reporting. For further information on Nature Research policies, see Authors & Referees and the Editorial Policy Checklist .

Statistics

For all statistical analyses, confirm that the following items are present in the figure legend, table legend, main text, or Methods section.

n/a	Confirmed	
☐	☒	The exact sample size (*n*) for each experimental group/condition, given as a discrete number and unit of measurement
☐	☒	A statement on whether measurements were taken from distinct samples or whether the same sample was measured repeatedly
☐	☒	The statistical test(s) used AND whether they are one- or two-sided<br>*Only common tests should be described solely by name; describe more complex techniques in the Methods section.*
☒	☐	A description of all covariates tested
☒	☐	A description of any assumptions or corrections, such as tests of normality and adjustment for multiple comparisons
☐	☒	A full description of the statistical parameters including central tendency (e.g. means) or other basic estimates (e.g. regression coefficient) AND variation (e.g. standard deviation) or associated estimates of uncertainty (e.g. confidence intervals)
☐	☒	For null hypothesis testing, the test statistic (e.g. *F*, *t*, *r*) with confidence intervals, effect sizes, degrees of freedom and *P* value noted<br>*Give P values as exact values whenever suitable.*
☒	☐	For Bayesian analysis, information on the choice of priors and Markov chain Monte Carlo settings
☒	☐	For hierarchical and complex designs, identification of the appropriate level for tests and full reporting of outcomes
☒	☐	Estimates of effect sizes (e.g. Cohen's *d*, Pearson's *r*), indicating how they were calculated

Our web collection on statistics for biologists contains articles on many of the points above.

Software and code

Policy information about availability of computer code

Data collection	no software used
Data analysis	Prism 8 and FlowJo 10

For manuscripts utilizing custom algorithms or software that are central to the research but not yet described in published literature, software must be made available to editors/reviewers. We strongly encourage code deposition in a community repository (e.g. GitHub). See the Nature Research guidelines for submitting code & software for further information.

Data

Policy information about availability of data

All manuscripts must include a data availability statement. This statement should provide the following information, where applicable:
- Accession codes, unique identifiers, or web links for publicly available datasets
- A list of figures that have associated raw data
- A description of any restrictions on data availability

X-ray crystallographic structure files of human STING-CTD were obtained from the Protein Data Bank (PDB) using accession codes: 4F5D and 4F5E. The datasets generated during and/or analysed during the current study are available from the corresponding author on reasonable request.

Field-specific reporting

Please select the one below that is the best fit for your research. If you are not sure, read the appropriate sections before making your selection.

☒ Life sciences ☐ Behavioural & social sciences ☐ Ecological, evolutionary & environmental sciences

October 2018

For a reference copy of the document with all sections, see nature.com/documents/nr-reporting-summary-flat.pdf

Life sciences study design

All studies must disclose on these points even when the disclosure is negative.

Sample size	No sample size calculations were performed for this study. All experiments had large effects and were repeatable and precise thus we determined that two biological replicates were sufficient. Where statistical tests were applied, we selected n=3 as the minimum sample size.
Data exclusions	No data was excluded
Replication	Individual experiments were replicated at least two times. All data replications were successful
Randomization	Not relevant for our study: only one variable is tested in each experiment
Blinding	Not relevant for our study: only one variable is tested in each experiment

Reporting for specific materials, systems and methods

We require information from authors about some types of materials, experimental systems and methods used in many studies. Here, indicate whether each material, system or method listed is relevant to your study. If you are not sure if a list item applies to your research, read the appropriate section before selecting a response.

Materials & experimental systems

n/a	Involved in the study	
☐	☒	Antibodies
☐	☒	Eukaryotic cell lines
☒	☐	Palaeontology
☒	☐	Animals and other organisms
☒	☐	Human research participants
☒	☐	Clinical data

Methods

n/a	Involved in the study	
☒	☐	ChIP-seq
☐	☒	Flow cytometry
☒	☐	MRI-based neuroimaging

Antibodies

Antibodies used

cGAS, Cell Signaling, 1:1000, D1D3G, #15102, Lot 1
beta-Actin, Cell Signaling, 1:1000, 8H10D10, #3700, Lot 15
Goat anti-rabbit, Licor, 1:10000, 926-3221, C40325-02
Goat anti-mouse, Licor, 1:10000, 926-68070, C60405-08

Validation

According to the manufacturer's information, the rabbit monoclonal anti-cGAS antibody (D1D3G, Cell Signaling Technology) is suitable for WB and reacts with human cGAS. The specificity of the antibody was tested by western blot using HEK 293T cells mock transfected or transfected with Myc-tagged full-length cGAS. For more information about this antibody: https://www.cellsignal.com/products/primary-antibodies/cgas-d1d3g-rabbit-mab/15102

According to the manufacturer's information, the rabbit monoclonal anti-β-Actin antibody (8H10D10, Cell Signaling Technology) is suitable for WB, IHC-P, IF-IC, and F and reacts with human, mouse, rat, hamster, monkey, and dog β-Actin. For more information about this antibody: https://www.cellsignal.com/products/primary-antibodies/b-actin-8h10d10-mouse-mab/3700?site-search-type=Products

Eukaryotic cell lines

Policy information about cell lines

Cell line source(s)	HEK293T cells were provided by D. Stetson (University of Washington, Seattle) and available through ATCC (ATCC® CRL-3216™).
Authentication	HEK293T cells were authenticated using Human STR Profiling Cell Authentication Service (ATCC).
Mycoplasma contamination	HEK293T cells were mycoplasma negative using PlasmoTest™ - Mycoplasma Detection Kit (InvivoGen)

Commonly misidentified lines (See ICLAC register)	None

Flow Cytometry

Plots

Confirm that:

- [x] The axis labels state the marker and fluorochrome used (e.g. CD4-FITC).
- [x] The axis scales are clearly visible. Include numbers along axes only for bottom left plot of group (a 'group' is an analysis of identical markers).
- [x] All plots are contour plots with outliers or pseudocolor plots.
- [x] A numerical value for number of cells or percentage (with statistics) is provided.

Methodology

Sample preparation	HEK 293T cell culture media was aspirated, replaced by PBS, and cells were dissociated using a P1000 and then analysed
Instrument	BD LSR II 334600 A 01/21/2005
Software	FlowJo 10
Cell population abundance	At least 25% of cells were used in final analysis. Stringent gates were chosen to ensure single cells were being analyzed.
Gating strategy	Gate 1 took the majority of the cells minus large clumps and debris, Gate 2 and 3 were both single cell selection gates, and Gate 4 was chosen to select the population of cells which linearly expressed both mTFP and mKO2 Flurophores

- [x] Tick this box to confirm that a figure exemplifying the gating strategy is provided in the Supplementary Information.
